# Supplementary material for: A Cyborg Insect Reveals a Function of a Muscle in Free Flight
Source: Cyborg Bionic Syst. 2022 May 4;2022:9780504. doi: 10.34133/2022/9780504 (PMC9494732; doi:10.34133/2022/9780504)
Supplement: Supplementary 1 — Figure S1: the flight speed of beetle with different loads. Figure S2: stimulation waveform and electrical current flow through the subalar muscle. Figure S3: EMG of nearby muscles during the electrical stimulation of the subalar muscle. [file 9780504.f1.pdf]

SUPPLEMENTARY MATERIALS

Supplementary Figures

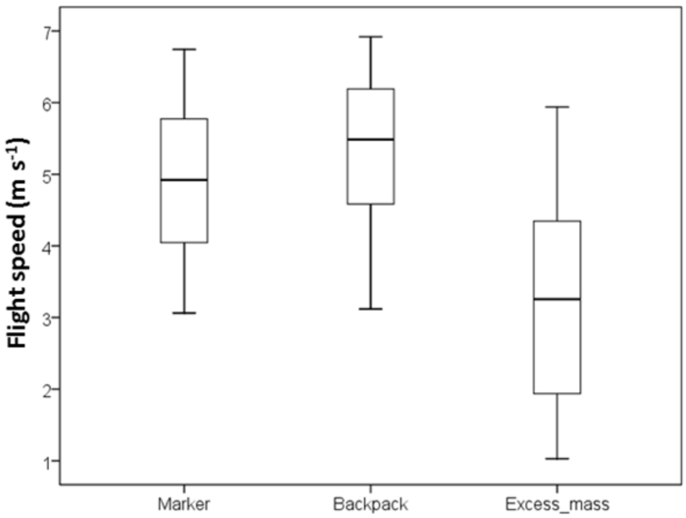

Fig. S1. The flight speed of beetle with different loads. The beetles experienced 3 different loading conditions (only marker mounted (0.25 g), backpack mounted (1.23 g) and excess mass mounted (3.50 g)) while flying freely in the flight arena. There is no difference in flight speed of the beetles mounted only small marker and those with backpack ($p>0.05$, paired samples t-test) while those mounted excess mass show clear reduction in flight speed ($p <0.001$, paired samples t-test) (N=4 beetles, n=60 trials).

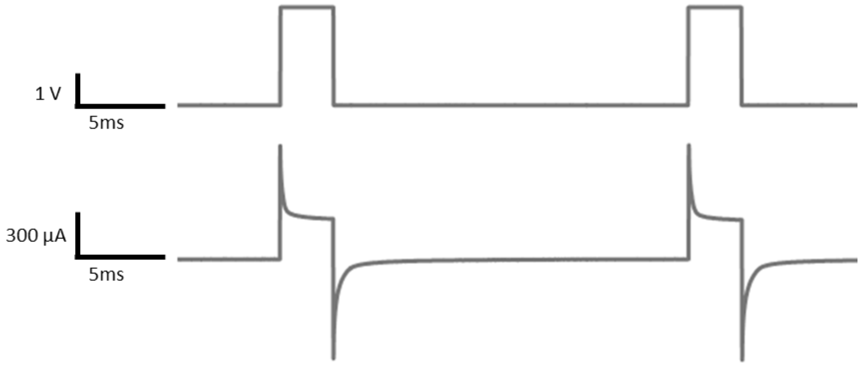

Fig. S2. Stimulation waveform and electrical current flow through the subalar muscle. (a) The stimulation pulse train (3V, 3 ms pulse width and 50 Hz) from the waveform function generator was applied on the subalar muscle via the two implanted electrodes. (b) The current waveform passed through the subalar muscle.

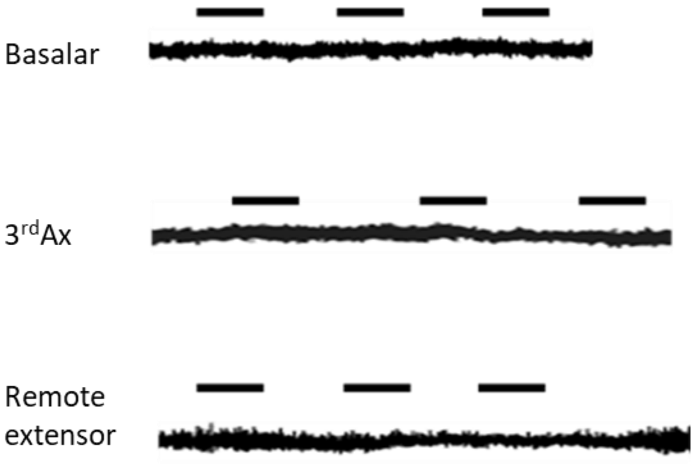

Fig. S3. EMG of nearby muscles during the electrical stimulation of the subalar muscle. One pair of silver wires was implanted into the subalar muscle for electrical stimulation another pair of silver wires was implanted to basalar muscle, 3^{rd}Ax muscle or remote extensor muscle for recording EMG signal. Except the nerves, all the hinges and joints were removed before recording to avoid mechanical interaction of the muscles. No EMG spikes observed in the nearby muscles while stimulating the subalar muscles ($N = 3$, $n = 60$, $p < 0.05$, binomial test).

Supplementary Movie

Movie S1. The response of the beetle due to electrical stimulation of subalar muscle. When both subalar muscles were stimulated, the beetle pitched and climbed up. It then recovered the body angle after the stimulation finished. The red and blue bars represent the body axis of the beetle with and without electrical stimulation, respectively.
